# Revealing the Specific Contributions of Mitochondrial CB_1_ Receptors to the Overall Function of Skeletal Muscle in Mice

**DOI:** 10.3390/cells14191517

**Published:** 2025-09-28

**Authors:** Zoltán Singlár, Péter Szentesi, Nyamkhuu Ganbat, Barnabás Horváth, László Juhász, Mónika Gönczi, Anikó Keller-Pintér, Attila Oláh, Zoltán Máté, Ferenc Erdélyi, László Csernoch, Mónika Sztretye

**Affiliations:** 1 Department of Physiology, Faculty of Medicine, University of Debrecen, 4032 Debrecen, Hungary; singlar.zoltan@med.unideb.hu (Z.S.); szentesi.peter@med.unideb.hu (P.S.); nyamkhuu.ganbat@med.unideb.hu (N.G.); gonczi.monika@med.unideb.hu (M.G.); olah.attila@med.unideb.hu (A.O.); csl@edu.unideb.hu (L.C.); 2 HUN-REN Cell Physiology Research Group, University of Debrecen, 4032 Debrecen, Hungary; 3 Doctoral School of Molecular Medicine, University of Debrecen, 4032 Debrecen, Hungary; 4 Department of Biochemistry, Albert Szent-Györgyi Medical School, University of Szeged, 6720 Szeged, Hungary; horvath.barnabas@med.u-szeged.hu (B.H.); keller.aniko@med.u-szeged.hu (A.K.-P.); 5 Centre of Excellence for Interdisciplinary Research, Development and Innovation, University of Szeged, 6720 Szeged, Hungary; 6 Institute of Surgical Research, Albert Szent-Györgyi Medical School, University of Szeged, 6720 Szeged, Hungary; juhasz.laszlo.1@med.u-szeged.hu; 7 Medical GeneTechnology Unit, HUN-REN Institute of Experimental Medicine, 1083 Budapest, Hungary; matez@koki.hu (Z.M.); erdelyi@koki.hu (F.E.)

**Keywords:** ATP, calcium homeostasis, cannabinoid receptor type 1, mitochondria, mitochondrial cannabinoid receptor type 1, murine skeletal muscle, muscle force, mtCB_1_ knockout, skeletal endocannabinoid system

## Abstract

Skeletal muscle, constituting 40–50% of total body mass, is vital for mobility, posture, and systemic homeostasis. Muscle contraction heavily relies on ATP, primarily generated by mitochondrial oxidative phosphorylation. Mitochondria play a key role in decoding intracellular calcium signals. The endocannabinoid system (ECS), including CB_1_ receptors (CB_1_Rs), broadly influences physiological processes and, in muscles, regulates functions like energy metabolism, development, and repair. While plasma membrane CB_1_Rs (pCB_1_Rs) are well-established, a distinct mitochondrial CB_1_R (mtCB_1_R) population also exists in muscles, influencing mitochondrial oxidative activity and quality control. We investigated the role of mtCB_1_Rs in skeletal muscle physiology using a novel systemic mitochondrial CB_1_ deletion murine model. Our in vivo studies showed no changes in motor function, coordination, or grip strength in mtCB_1_ knockout mice. However, in vitro force measurements revealed significantly reduced specific force in both fast-twitch (EDL) and slow-twitch (SOL) muscles following mtCB_1_R ablation. Interestingly, knockout EDL muscles exhibited hypertrophy, suggesting a compensatory response to reduced force quality. Electron microscopy revealed significant mitochondrial morphological abnormalities, including enlargement and irregular shapes, correlating with these functional deficits. High-resolution respirometry further demonstrated impaired mitochondrial respiration, with reduced oxidative phosphorylation and electron transport system capacities in knockout mitochondria. Crucially, mitochondrial membrane potential dissipated faster in mtCB_1_ knockout muscle fibers, whilst mitochondrial calcium levels were higher at rest. These findings collectively establish that mtCB_1_Rs are critical for maintaining mitochondrial health and function, directly impacting muscle energy production and contractile performance. Our results provide new insights into ECS-mediated regulation of skeletal muscle function and open therapeutic opportunities for muscle disorders and aging.

## 1. Introduction

Making up 40–50% of total body mass, skeletal muscles are essential for overall health. They not only maintain posture and enable mobility but also actively regulate homeostasis and metabolism of the whole body [1,2,3]. Muscle contraction and force generation result from the sequence of events known as excitation–contraction coupling (ECC) [4,5]. Muscle contraction and force generation require the continuous availability of chemical energy in the form of ATP. Mitochondria, which constitute approximately 10–15% of muscle fiber volume, are the primary sites for ATP production via oxidative phosphorylation (OXPHOS) [6]. Beyond energy provision, mitochondria are instrumental in decoding intracellular Ca^2+^ signals, thereby contributing significantly to the spatio-temporal regulation of Ca^2+^ concentration [7,8,9,10]. The integrity of mitochondrial morphology and its dynamic nature are crucial for effective ATP synthesis, enabling the maintenance of the mitochondrial membrane potential (*ΔΨ_m_*).

The endocannabinoid system (ECS) is a complex signaling network essential for various physiological processes, including, but not limited to mood, emotion, pain modulation, immune responses, cutaneous homeostasis, appetite, and metabolism [11,12,13,14,15]. Its core components are the “classical” cannabinoid receptors (CB_1_R and CB_2_R), endocannabinoids, and the enzymes and transport systems governing their synthesis and degradation [15,16,17,18].

In skeletal muscles, CB_1_Rs (encoded by the *Cnr1* gene) are more abundant than CB_2_Rs, and regulate various cellular functions, such as autophagy [19], energy metabolism [20,21], muscle development [22], inflammation, and repair [23]. CB_1_Rs influence biochemical processes like ATP production and reactive oxygen species (ROS) modulation [24]. Evidence suggests that CB_1_ activation inhibits myotube formation and the differentiation of primary human satellite cells, i.e., a process that is critical for muscle regeneration [25,26]. Conversely, CB_1_ knockout embryos show an increased number of muscle fibers, and postnatal mice display larger muscle fiber diameters, indicating that CB_1_ typically exerts an inhibitory effect on myogenesis. CB_1_ antagonism has also been shown to activate the Akt-mammalian target of rapamycin (mTOR) axis, which stimulates cell growth and protein synthesis in skeletal muscle tissue when sufficient energy is present [27,28,29]. On the other hand, CB_1_ receptor over-activation was observed in, e.g., Duchenne Muscular Dystrophy (DMD) and metabolic conditions such as obesity [30,31,32,33,34]. Indeed, CB_1_Rs appear to play a complex role in DMD pathophysiology, detrimentally affecting muscle regeneration, mitochondrial health, and inflammation [34]. Consequently, CB_1_ receptor antagonism is being explored as a therapeutic strategy to improve metabolic health and reduce body fat [35]. A recent study demonstrated that global deletion of CB_1_Rs in mouse *m. gastrocnemius* induced a fast-to-slow twitch fiber-type conversion, enhancing its oxidative capacity and influencing antioxidant defense systems [32]. CB_1_ activation can also suppress the expression of the pro-inflammatory cytokine IL-6 in skeletal muscle [36]. Genetic manipulation of CB_1_Rs, such as a muscle-specific knockdown, has been shown to result in reduced muscle force generation in vivo and in vitro [37,38].

Beyond the well-known plasma membrane-localized peripheral CB_1_ (pCB_1_R) [39,40], a distinct population of mitochondrial CB_1_ receptors (mtCB_1_R) exists in muscles and in other tissues [31,41,42,43,44]. These mtCB_1_Rs were shown to suppress mitochondrial activity and are thought to play a role in mitochondrial quality control, including biogenesis, fusion/fission dynamics, mitophagy, and the mitochondrial unfolded protein response [45]. Previous research proposed a correlation between upregulated endocannabinoid effects and mitochondrial dysfunction leading to neurodegenerative disorders [46]. Activation of mtCB_1_ is known to decrease cAMP production, consequently reducing PKA and Complex I activity [31,42]. Therefore, mtCB_1_ receptors are hypothesized to depress mitochondrial respiration and potentially enhance cannabinoid-mediated physiological responses [47], such as affecting short-term plasticity of GABAergic neurotransmissions in the nervous system [48]. Indeed, a growing body of evidence suggests that mtCB_1_ receptor activation can directly influence the electron transport chain and oxidative phosphorylation [31,32,39,44,49], thereby impacting the cell’s energy output and overall mitochondrial homeostasis. For example, in the brain, mtCB_1_ activation has been shown to reduce mitochondrial respiration and ATP production [31,42,50].

In the current study we created a global mitochondrial CB_1_ receptor mouse model. By using a combination of genetic and pharmacological approaches, we report a key role of mtCB_1_Rs in skeletal muscle physiology. Our findings offer novel insights into the complex involvement of mtCB_1_Rs in ECS-mediated regulation of skeletal muscle contraction, force generation, mitochondrial morphology, and energetics.

## 2. Materials and Methods

### 2.1. Animal Care

This study was conducted in strict accordance with the ethical guidelines of the European Community (86/609/EEC), with all efforts made to reduce animal suffering and distress. The experimental protocol received full approval from the University of Debrecen’s Institutional Animal Care Committee (4-2/2024/DEMÁB). The mice were housed in optimal conditions, including plastic cages with mesh covers, unrestricted access to food and water, and a controlled room environment with a 12 h light/12 h dark cycle at 22–25 °C.

### 2.2. Generation of the mtCB_1_-KO Mouse Strain

mtCB_1_-KO mice (C57BL/6NTac:*Cnr1^Em1Δ1^-^21/EMGTU^*), referred to hereinafter as mtCB_1_^−/−^, were generated in Medical GeneTechnology Unit, HUN-REN Institute of Experimental Medicine (Budapest, Hungary). Briefly, a 21-amino acid deletion immediately downstream of the ATG start codon in the CB_1_ receptor gene (responsible for the mitochondrial targeting as previously described in [39]) was introduced using CRISPR/Cas9-mediated genome editing. Two single guide RNAs were designed to target Cas9 cleavage sites flanking the deletion region, ensuring that the recognition sequences were removed to prevent re-cutting. A single-stranded oligodeoxynucleotide (ssODN) template containing the desired deletion was used to facilitate homology-directed repair (HDR). Cas9 protein (30 ng/µL), sgRNAs (15 ng/µL each), and the ssODN donor (15 ng/µL) were co-injected into the pronuclei of in vitro fertilized C57BL/6 embryos. Resulting pups were genotyped by PCR using primers flanking the deletion, yielding a 251 bp product for the wild-type allele and a 188 bp product for the deletion allele. A founder carrying the deletion allele was identified, and the target region was cloned and sequenced to confirm the deletion.

Following the above-described, N-terminal deletion of 21 amino acids (63 base pairs) in the mouse CB_1_-receptor coding sequence, the theoretical probability of mitochondrial CB_1_ expression is reduced from 40–45% to 1–3% [39]. After multistep crossing and genotyping, homozygous mtCB_1_^+/+^ and mtCB_1_^−/−^ mice were generated in the animal house of the Department of Physiology (University of Debrecen). For the current experiments, mixed sex 3–4-month-old homozygous mtCB_1_^+/+^ and mtCB_1_^−/−^ animals were used.

### 2.3. Genotyping

Mice were genotyped at 3 to 4 weeks of age to classify them into experimental groups. This was done by isolating total genomic DNA from finger biopsies and screening for the presence of the gene encoding the mitochondrial CB_1_R using a previously described PCR method [37].

### 2.4. Wire Hang Test

Both mtCB_1_^−/−^ and mtCB_1_^+/+^ mice were tested for their muscular strength in the wire hang test. Mice were placed on a horizontal wire, which they grasped with their forelimbs. The duration (in seconds) for which each mouse was able to maintain its grip was recorded. The trial ended when the mouse released its grip and fell onto a padded surface (a litter-filled box) positioned below to ensure a safe landing. This sequence of events was repeated 5 times, and the average was noted for each animal.

### 2.5. Grip Strength Test

As previously described [51], grip force was measured when animals were gently dragged away from the grip test meter, following a firm grab of its bar for a brief period of time. Before the animal released the bar, an online connected computer recorded the maximum force and digitized it at a frequency of 2 kHz. To get an averaged single data point, each animal underwent the test ten to fifteen times. Data points were then normalized to the measured bodyweight. 6 mixed gender animals were tested for both genotypes and no differences in bodyweight were noted.

### 2.6. Rota-Rod Test

Mice were tested on a Rota-Rod device (cat.no. YPFB00, Dev Scientific and Engineering, India), and the latency to fall was recorded in seconds. The device operated on an accelerating protocol, with the speed increasing from 4 to 300 rpm over 300 s. For acclimatization, mice completed three one-minute trials at a constant speed of 4 rpm, with 10 min intervals. After a 30 min rest, the accelerating protocol was performed in three trials. The final score for each mouse was the average of these three trials.

### 2.7. In Vitro Experiments

Mice were sacrificed in compliance with the guidelines of the European Community (86/609/EEC). After cervical dislocation, *flexor digitorum brevis* (FDB), *extensor digitorum longus* (EDL), *tibialis anterior* (TA), *soleus* (SOL), and *gastrocnemius* (GA) muscles from the hind limb were dissected manually under a transmitted light microscope using thin forceps and fine precision surgical scissors. In all subsequent experiments, the muscle types used are noted in the respective section.

#### 2.7.1. Isometric Force Measurement

Muscle contractions were measured in accordance with our earlier reports [52,53]. Briefly, Krebs solution (containing in mM: 135 NaCl, 5 KCl, 2.5 CaCl_2_, 1 MgSO_4_, 10 Hepes, 10 glucose, 10 NaHCO_3_; pH 7.2; room temperature) was continuously superfused (10 mL/min) onto fast- and slow-twitch muscles (EDL and SOL, respectively) positioned horizontally in an experimental chamber that was equilibrated with 95% O_2_ plus 5% CO_2_. Muscles were connected to a capacitive mechano-electric force transducer (Experimetria, Budapest, Hungary) at one end, with the other restrained to a pin. To induce single twitches, two-millisecond supramaximal pulses were delivered via two platinum electrodes positioned below the muscle. Axotape software (version 1.2.01, Axon Instruments, Foster City, CA, USA) was used to store the force responses after being digitized at a frequency of 2 kHz using a Digidata 1200 A/D card. The transducer was then moved to a length that produced the maximum force response, stretching the muscles and allowing them to equilibrate for six minutes. Single pulses elicited single twitches at 0.5 Hz. Under these circumstances, a minimum of ten twitches from each muscle were recorded. If the amplitudes of the individual force transients inside such a train varied by less than 3%, the muscle was included in the experiment. Single pulses with frequencies of 200 Hz for 200 ms (EDL) or 100 Hz for 500 ms (SOL) were used to induce tetanus. The time interval between the start of the transient and the relaxation to 10% of peak force was used to calculate the durations of individual twitches and tetani.

Using 200-ms-long isometric contractions at 20, 40, 60, 80, 100, 120, 160, and 200 Hz with 30 s intervals, the force–frequency relationship in EDL was determined. The protocol used in SOL was 500 ms long at 10, 20, 30, 40, 60, 80, and 100 Hz.

#### 2.7.2. Isolation of Single FDB Fibers

Single muscle fibers from FDB muscles were enzymatically dissociated in a calcium-free Ringer solution that contained 0.2% Type I collagenase (cat. no. 17100-017, Gibco, Grand Island, NY, USA) at a temperature of 37 °C for a duration of 45–50 min. To obtain single fibers, the FDB muscles underwent mechanical dissociation followed by gentle trituration in a standard Ringer solution (composed of mM: 2.6 CaCl_2_, 136 NaCl, 5 KCl, 1 MgCl_2_, 10 Hepes; 10 glucose; pH 7.2). The isolated fibers were subsequently placed in culture dishes and stored at 4 °C in the refrigerator for a maximum of 36 h before use. Only those fibers exhibiting clearly visible striations, without any swelling or damage to the surface membrane, were chosen for further experiments.

#### 2.7.3. Immunofluorescent Labeling and Super-Resolution Microscopy

For stimulated emission depletion (STED) microscopy, following enzymatic digestion and trituration, FDB fibers were probed with 250 nM Abberior LIVE Orange mito fluorescent probe (cat.no. LVORANGE-0146-30NMOL, Göttingen, Germany) (60 min, 37 °C) for specific staining of the mitochondrial cristae. Then, fibers were fixed with 4% paraformaldehyde solution for 15 min. Following phosphate-buffered saline (PBS) washing, 0.25% Triton X-100 was used for permeabilization. After applying a blocking solution (2% BSA, 0.1% Tween20 in PBS), cells were incubated with anti-CB_1_R antibody (designed to recognize the C-terminal [unmodified] region of the receptor) overnight at 4 °C (1:500 in the above blocking solution, Thermo-Fisher Scientific, Waltham, Massachusetts, USA, cat.no. PA5-85080). Samples were washed with PBS post-incubation. For the secondary antibody treatment, STED-compatible Abberior STAR Red (anti-rabbit, 1:300 in the above blocking solution, cat. no. STRED-1002-20UG, Göttingen, Germany) was used. Following PBS washing, a specific liquid mounting medium was applied on prepared samples (Abberior MOUNT, LIQUID ANTIFADE, cat.no. MM-2009-2X15ML, Göttingen, Germany).

For microscopy, the STEDYCON system (Abberior, Göttingen, Germany) combined with an inverted Zeiss Axiovert 135 microscope (Zeiss, Oberkochen, Germany) was employed. 64 µm pinhole size and a 100x oil immersion objective were set for imaging. The excitation wavelength of Abberior STAR RED was 640 nm, and for Abberior LIVE Orange mito 561 nm was used. A depletion wavelength of 775 nm was applied for STED, with detection wavelengths set to 660 and 616 nm, respectively.

An arbitrary ROI (marked with a white rectangle) was selected always in parallel with the longitudinal axis of the fiber, and the fluorescence was plotted. The ROI selection was done in a way to avoid areas of dye accumulation (e.g., nuclei). A total of 19 and 10 arbitrary ROIs, respectively, from 5-5 STED images were analyzed for each genotype (mtCB_1_^+/+^ and mtCB_1_^−/−^, *N* = 2 animals/group). Fluorescence trace was calculated by averaging the lines parallel with the fiber axis along the chosen rectangle. As a next step, the distance between the neighboring peaks of the curve was assessed, and from all ROIs we created a histogram distribution of these distances. Finally, this distribution was fitted with a Gaussian function according to Equation (1) below:

(1) $$y=ae^{\left[-0.5{\left(\frac{x-x_{0}}{b}\right)}^{2}\right]}$$

where a is the amplitude (or height) of the peak, x_0_ is the mean (or center) of the distribution, and b is the standard deviation, which determines the width of the Gaussian curve.

#### 2.7.4. Mitochondrial Membrane Potential Measurement, Confocal Microscopy, and Image Processing

Isolated FDB fibers from mtCB_1_^+/+^ and mtCB_1_^−/−^ mice were incubated with 20 nM tetra-methylrhodamine methyl ester (TMRE, cat.no. 87917-25MG, Merck, Darmstadt, Germany) in standard Ringer’s solution at ambient temperature for a duration of 15 min. Following this, the surplus dye was removed after 20 min. To assess TMRE fluorescence, which serves as an indicator of the mitochondrial membrane potential (*Δψ_m_*), a series of images was captured over time using a Zeiss 5 Live confocal microscope (Zeiss, Oberkochen, Germany) fitted with a 20× air objective and a 543 nm laser. The pinhole setting was adjusted to 55 µm. Sequential imaging was conducted at intervals of 10 s. At 40 s into the imaging series, 1 μM FCCP (cat.no. C2920, Merck, Darmstadt, Germany) was introduced to the fiber, while the decline in TMRE fluorescence, reflecting the dissipation of the *Δψ_m_* was continuously observed. Utilizing the Zeiss Zen Blue analyzer software (version 3.12, Zeiss, Oberkochen, Germany), a region of interest (ROI) within the fiber was designated on all subsequent images of the recorded series, and the average fluorescence values obtained over time were fitted to an exponential function using Equation 2 to ascertain the fluorescence decay time (τ). The averages of the individual decay times were then plotted.

The exponential decay of the *Δψ_m_* was assessed by the equation below:

(2) $$y=y_{0}+ae^{-bx}$$

where 1/b is the decay time (τ) expressed in seconds.

#### 2.7.5. Evaluation of Mitochondrial Morphology Using Transmission Electron Microscopy

The isolated TA muscles were subjected to treatment with a fixative solution consisting of 3% glutaraldehyde in Millonig’s buffer. Subsequently, small bundles of the fixed fibers underwent post-fixation using 1% OsO_4_ in water. The specimens were rapidly dehydrated through a series of graded ethanol solutions, followed by an intermediate step with propylene oxide. The dehydrated samples were then embedded in Durcupan epoxy resin (cat.no. 44610-1EA, Sigma-Aldrich, Burlington, MA, USA). Ultrathin horizontal sections were prepared using a Leica Ultracut UCT ultramicrotome (Leica Microsystems, Vienna, Austria) and subsequently stained with uranyl acetate and lead citrate. The sections were examined with a JEM1010 transmission electron microscope (JEOL, Tokyo, Japan), which was equipped with an Olympus camera. Longitudinally oriented micrographs were captured at a consistent magnification of 20,000× and a similar horizontal field width of 7.3 µm, and these images were analyzed using ImageJ software (version 1.53k, Java 1.8.0_172, NIH, Bethesda, MD, USA). The area and perimeter of the mitochondria were the primary parameters of interest.

#### 2.7.6. Mitochondrial Calcium Uptake Measurement

Changes in mitochondrial calcium levels within individual FDB fibers after repetitive electrical stimulation were observed using rhod-2, a calcium-sensitive dye, in accordance with the methodology established by Ainbinder and colleagues [54]. FDB fibers were treated with 5 µM rhod-2-AM for 15 min at ambient temperature, followed by a wash with a dye-free normal Tyrode’s solution. The fibers underwent electrical stimulation (S88 Stimulator, Grass Technologies, Warwick, RI, USA) via a pair of platinum electrodes positioned near the fiber of interest. A single tetanus or a series of five consecutive tetani (500 ms duration, 100 Hz) were administered at a supramaximal activating voltage for each cell. Time series x-y images (512 × 512 pixels, 0.5 ms/pixel) were captured at rest, after the first and fifth tetanus, and during a 10 min recovery period post the final stimulation. The calculation of rhod-2 fluorescence values derived from the mitochondria (F_mito_) was executed as previously described [53]. In summary, fluorescence was measured at the peaks (I-band fluorescence, indicative of mitochondria (F_I-band_)) and at the troughs (A-band fluorescence, representing baseline (F_A-band_)), followed by the computation of normalized mitochondrial fluorescence expressed as F_mito_, which reflects the normalized mitochondrial calcium uptake, using the equation provided below:

(3) $$F_{mito}=\frac{F_{I\text{-}band}-F_{A\text{-}band}}{F_{A\text{-}band}}$$

#### 2.7.7. Assessment of Mitochondrial Oxygen Consumption Using High-Resolution Respirometry

Mitochondrial oxygen consumption (O_2_ flux) was assessed in GA muscle fibers (10 mg/chamber) using High-Resolution FluoRespirometry (Oxygraph-2k, Oroboros Instruments, Innsbruck, Austria). The isolated GA muscle fibers were incubated in BIOPS solution containing saponin to ensure membrane permeabilization. After stabilization of baseline respiration, we used a short and a long respirometric protocol (see below). All compounds used during these protocols were purchased from Sigma-Aldrich (Burlington, MA, USA).

In the short respirometric protocol, the integrity of the outer mitochondrial membrane was tested with exogenous cytochrome c [CytC] (10 µM) following Complex II-linked oxidative phosphorylation (OXPHOS II) stimulation (0.5 µM Rotenone [Rot], cat no. R8875; 10 mM succinate [S], cat.no. W327700; and 2.5 mM ADP, cat.no. 01905). Respiratory control ratio (RCR), an index of respiration coupled to ADP-ATP conversion, is expressed as a ratio of OXPHOS II to the ATP synthase-inhibited (oligomycin, Omy; 2.5 μM, cat.no. 495455) LEAK state. The electron transport system-independent respiration (or residual oxygen consumption; ROX) was determined after Complex III inhibition with antimycin A (2.5 μM, cat.no. A8674).

In the long respirometric protocol, Complex I-linked oxidative phosphorylation (OXPHOS I) was measured in the presence of Complex I-linked substrates (10 mM glutamate and 2 mM malate, cat.no. G1626 and M1000, respectively) and ADP (2.5 mM, cat.no. A5285). Rotenone (Rot; 0.5 µM, cat.no. R8875) was used to (a) inhibit complex I and (b) assess OXPHOS II in the presence of succinate (S; 10 mM, cat.no. S2378) and adenylate. After inhibition of Complex III (antimycin A; 2.5 µM, cat.no. A8674), Complex IV respiratory activity was measured with ascorbate (2 mM, cat.no. A7631) and artificial substrate N,N,N’,N’-Tetramethyl-p-phenylenediamine dihydrochloride (TMPD; 0.5 mM, cat.no. T3134). Ascorbate was added before TMPD to avoid uncontrollable autoxidation of the electron donor. Sodium azide (NaN_3_; 100 mM, cat.no. S2002) was finally administered to block Complex IV-linked mitochondrial respiration.

Both the short and long protocol measurements were performed in a Mir05 respiration medium under continuous magnetic stirring (750 rpm) at 37 °C. DatLab software (version 7.4.0.4, Oroboros Instruments, Innsbruck, Austria) was used for online display, respirometry data acquisition, and analysis.

### 2.8. Quantification and Statistical Analysis

Pooled data are expressed as mean ± standard deviation (SD) or error of the mean (SEM). The differences between mtCB_1_^+/+^ and mtCB_1_^−/−^ mice were assessed using Student’s t-test (paired, two-tailed). A *p*-value of less than 0.05 was considered statistically significant. The normality of the data was always tested with the Kolmogorov–Smirnov normality test in GraphPad Prism 8.0.1 (GraphPad Software, LLC, San Diego, CA, USA).

## 3. Results

### 3.1. The Targeted Deletion of Mitochondrial CB_1_ Receptors Alters Subcellular Localization and Spatial Distribution in Skeletal Muscle

**Figure 1:**
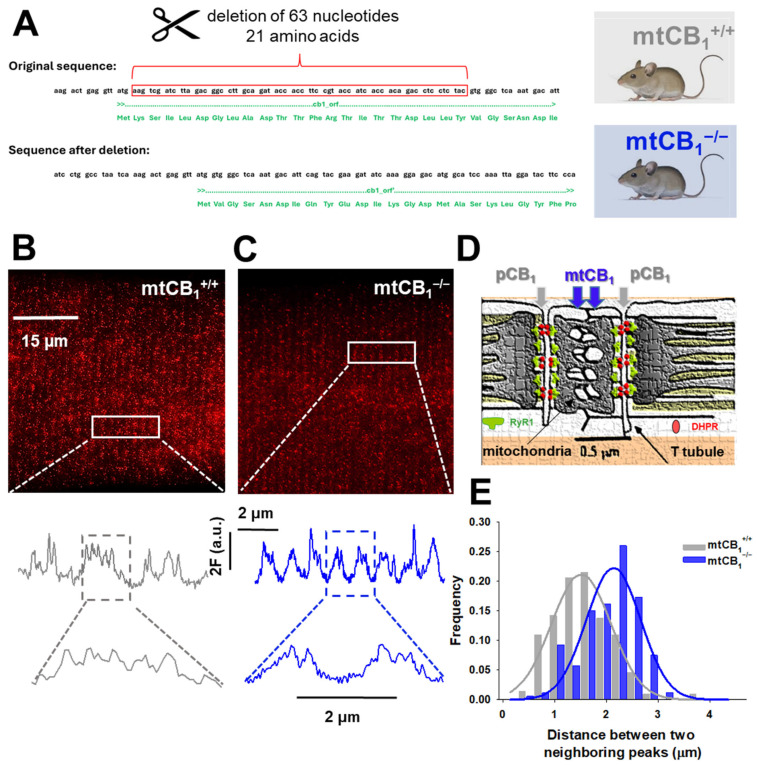
**Genetic engineering of mtCB_1_-KO mice.** (**A**) Deletion of 63 nucleotides from the original sequence of *Cnr1* leads to systemic loss of mitochondrial CB_1_ targeting (here and after referred to as mtCB_1_^−/−^). The mtCB_1_^+/+^ animals serve as controls (created with Biorender.com). (**B**,**C**) Deletion of mtCB_1_ was confirmed by STED microscopy. Zoomed-in regions (rectangles) show the Abberior STAR red-tagged CB_1_ antibody fluorescence distribution (expressed in a.u.). Note that in mtCB_1_^+/+^, more than two peaks per sarcomere are detected (fluorescence originates from both pCB_1_ and mtCB_1_ populations), whereas in mtCB_1_^−/−^ samples, two peaks per sarcomere are detected (fluorescence originates solely from the pCB_1_ population). (**D**) Cartoon illustrating the arrangement of two distinct CB_1_ populations in skeletal muscle cells: in the plasma membrane (pCB_1_) and the mitochondrial membrane (mtCB_1_), respectively (adapted from Pouvreau et al. 2007 [55]). (**E**) Histogram depicting the rightward shift of peak fluorescence distribution frequencies in mtCB_1_^−/−^ samples clearly underlying the lack of mitochondrial CB_1_ signals. A Gaussian was fit to the data set for mtCB_1_^+/+^ and mtCB_1_^−/−^ respectively, with the following parameters: a = 0.21 *vs*. 0.22; x_0_ = 0.57 vs. 0.52 and b = 1.51 vs. 2.14, where a is the amplitude and b depicts the width of the Gaussian curve fit.

Cannabinoids can activate CB_1_Rs localized at the plasma or mitochondrial outer membrane, but the role of specific subcellular pools of CB_1_ receptors in physiological muscle function is unknown. Since our laboratory has long been interested in studying the role of ECS in skeletal muscle [37,38,54], here, to obtain chronic mitochondrial deletion of the CB_1_ receptor, we followed a similar engineering strategy as described by Hebert-Chatelain and colleagues (2016) [39] (Figure 1A). Homozygous, mixed sex animals were employed in this study. The mitochondrial CB_1_ deletion in skeletal muscles was confirmed with super-resolution STED microscopy (Figure 1B,C). The traces from the zoomed-in regions recorded on single FDB cells from both genotypes clearly show the differential spatial distribution of the fluorescence signal. Consistent with the subcellular localization of CB_1_ receptors in muscle, namely, plasma (pCB_1_) and mitochondrial membranes (mtCB_1_) (Figure 1D), as one would expect, the double-row pattern with virtually parallel lines along the length of the T-tubule is seen in both specimens. Yet, the absence of mitochondrial CB_1_ receptors results in a distinct alteration of signal distribution, specifically, a marked reduction in staining between the double rows. This observation aligns with expectations, as the fluorescence originating from mitochondria within the cytoplasm would be lost following receptor deletion. To quantify this change, the longitudinal distance–i.e., parallel to the muscle fiber axis–between adjacent CB_1_ receptor signals was measured on several fibers and plotted as a distribution (Figure 1E). In the mtCB_1_-knockout samples, a rightward shift in the distribution indicates a significant increase in the spacing between neighboring receptors. This shift provides strong evidence that the mitochondrial CB_1_ receptors were lost, which directly contributed to the observed spatial reorganization of CB_1_ signals in mtCB_1_^−/−^ muscles.

### 3.2. The Lack of Mitochondrial CB_1_ Does Not Affect Coordination and Force Generation in Vivo

**Figure 2:**
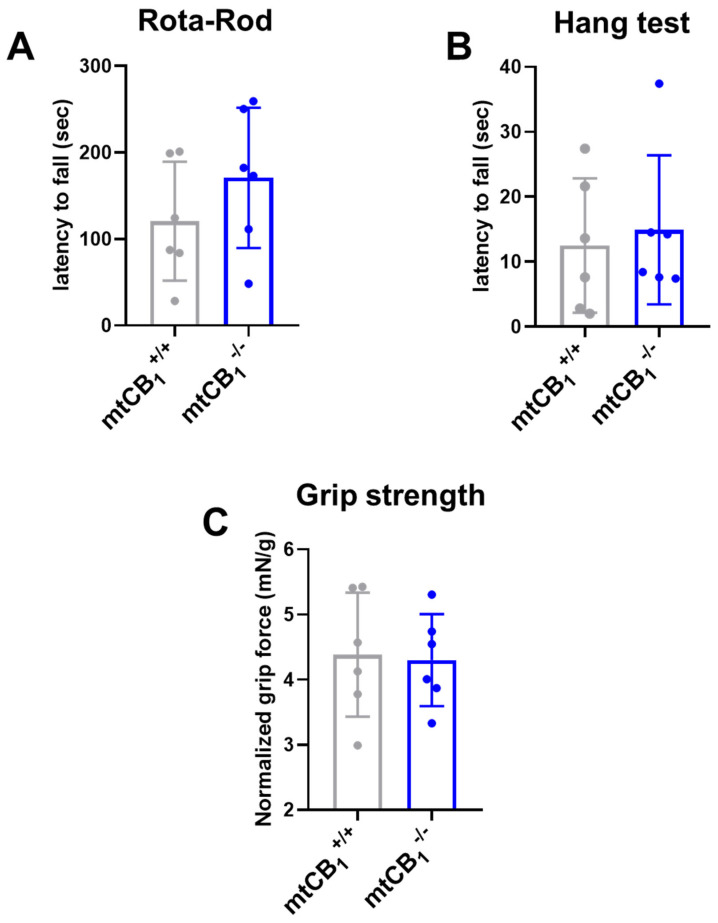
**Examination of in vivo coordination and muscle strength following mtCB_1_ deletion.** (**A**) Rota-Rod coordination tests revealed no change in the latency to fall following mtCB_1_ ablation. (**B**) Similarly, the wire hang test uncovered no change in the latency to fall in mtCB_1_^−/−^ specimens. (**C**) Grip strength normalized to body weight was preserved in mtCB_1_^−/−^ mice. Bar graphs represent mean ± SD for *N* = 6 mice/group in each test.

The mitochondrial KO strain is viable and fertile, and it presents normal locomotor activity (Figure 2A), muscular strength, as assessed by the wire hang test (Figure 2B), and grip force (Figure 2C). Interestingly, the systemic mtCB_1_-KO mice did not show any alteration in the in vivo tests, whereas the global CB_1_-KO mice and the skeletal muscle-specific inducible CB_1_-KO display a clear impairment [37,38,54,56].

### 3.3. The Suppression of Mitochondrial CB_1_ Expression Leads to Smaller Force Generation in Vitro

**Figure 3:**
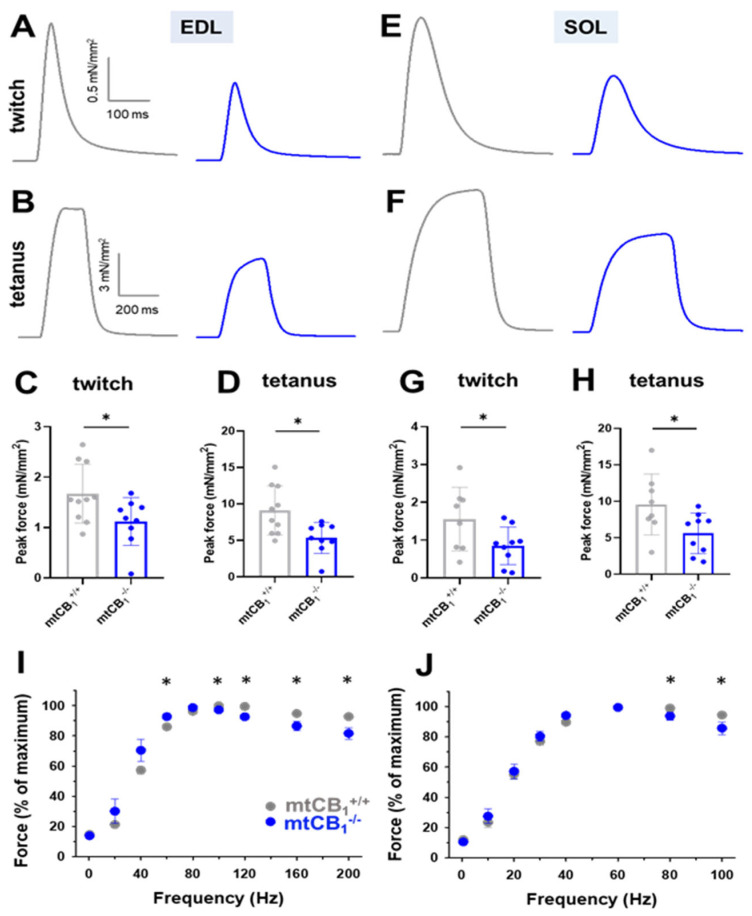
**Both fast and slow-twitch muscles of mtCB_1_^−/−^ mice exert decreased isometric force in vitro**. Representative ex vivo twitch (**A**,**E**) and tetanic force (**B**,**F**) on EDL and SOL muscles from mtCB_1_^+/+^ and mtCB_1_^−/−^ mice at room temperature (24 °C). Average twitch (**C**,**G**) and tetanic (**D**,**H**) force were normalized to the cross-sectional area of the given muscle. * shows a significant difference compared to the age-matched mtCB_1_^+/+^ at *p* < 0.05. Bar graphs show mean ± SD. Force–frequency relationship in EDL (**I**) and in SOL (**J**) muscles from mtCB_1_^+/+^ and mtCB_1_^−/−^ mice. Note that the mtCB_1_^−/−^ EDL muscles (panel **I**) fatigued earlier after reaching the maximum force output. *N* = 5 animals/ genotype. * *p* < 0.05.

To further explore the consequences of deleting mitochondrial CB_1_ on muscle performance, we conducted detailed in vitro force analyses. We examined both fast-twitch glycolytic muscles (EDL) and slow-twitch oxidative muscles (SOL), from both genotypes. Our analysis of representative twitch and tetanic forces demonstrated a marked decrease in peak force in both EDL (Figure 3A–D) and SOL muscles (Figure 3E–H). Moreover, when assessing the force–frequency relationship, we observed a tendency for mtCB_1_^−/−^ muscles to fatigue significantly quicker after reaching the maximum force output (80 Hz in EDL and 60 Hz in SOL, Figure 3I,J). In addition, in mtCB_1_^−/−^ EDL muscles, we observed improved excitability, which is notable at lower frequencies (20, 40, and 60 Hz, Figure 3I). This phenomenon was not seen in the SOL muscles. Table 1 and Table 2 provide a comprehensive overview of these experimental results for the two muscle types. Intriguingly, the EDL muscles of knockout (mtCB_1_^−/−^) specimens were significantly larger, weighing an average of 16.74 ± 0.45 mg compared to 13.67 ± 0.85 mg for mtCB_1_^+/+^ mice. This difference implies a larger cross-sectional area (CSA) in the knockout specimens (0.99 ± 0.08 mm^2^ for mtCB_1_^+/+^ vs. 1.21 ± 0.05 mm^2^ mtCB_1_^−/−^).

### 3.4. Suppression of Mitochondrial CB_1_ Leads to Altered Mitochondrial Morphology

**Figure 4:**
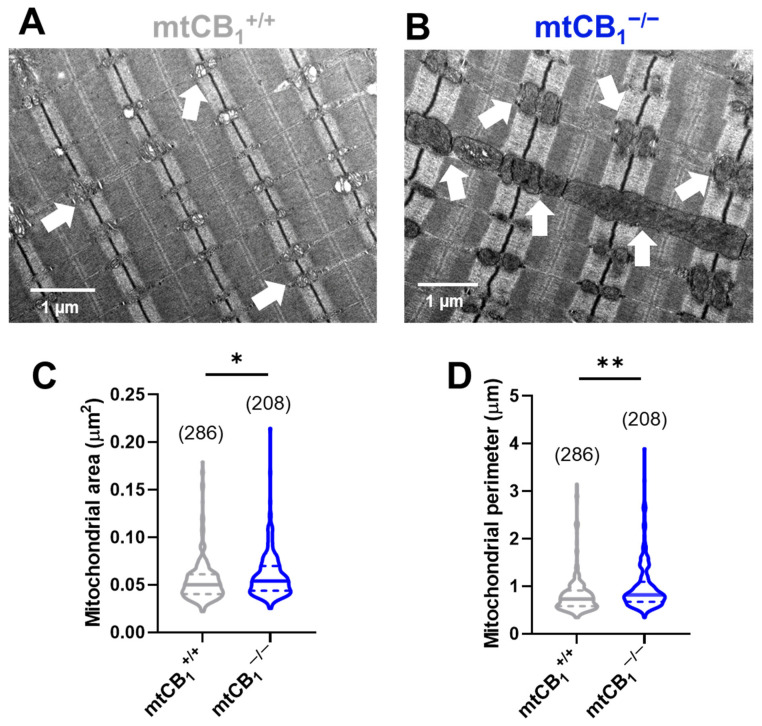
**Mitochondrial morphology is significantly altered in TA muscles of mtCB_1_^−/−^ mice.** Morphological analysis of the individual mitochondria was performed using TEM. Representative electron micrographs of myofibrils from longitudinal sections obtained on TA muscle samples from mtCB_1_^+/+^ (**A**) and mtCB_1_^−/−^ (**B**) illustrate drastically altered mitochondrial morphology. The white arrows point to mitochondria that are placed at the I band next to triads, always on the side closer to the Z line. Mitochondrial area (**C**) and perimeter (**D**) were determined from similar TEM images as shown in panels (**A**,**B**). The violin plots demonstrate the median (horizontal line) and the 25th (lower whisker) and 75th (upper whisker) percentiles. The numbers in parentheses indicate the number of analyzed mitochondria that originated from *N* = 3 mice. * *p* < 0.05, ** *p* < 0.001.

To investigate potential structural changes in the mitochondrial network of adult skeletal muscle fibers due to reduced mitochondrial CB_1_ expression, we used transmission electron microscopy (TEM) on TA muscles. We captured TEM images from longitudinal muscle sections of both mtCB_1_^+/+^ and mtCB_1_^−/−^ animals (representative images are shown on Figure 4A,B). Both images display the characteristic highly organized structure of skeletal muscles, with visible sarcomeres. In the mtCB_1_^+/+^ sample, the mitochondria (indicated by white arrows) appear relatively small, numerous, and uniformly distributed within the muscle fibers, typically nestled between the myofibrils (Figure 4A). Their internal structure (cristae) appears intact and well-defined. In the mtCB_1_^−/−^ sample, the mitochondria appear significantly larger, more swollen, and often irregularly shaped compared to the wild-type. To characterize these morphological changes, we quantified the area (0.05 ± 0.004 vs. 0.07 ± 0.004 *, *p* < 0.05, Figure 4C) and perimeter (0.84 ± 0.02 vs. 0.98 ± 0.03 **, *p* < 0.001, Figure 4D) of each mitochondrion identified in all TEM micrographs. Our analysis revealed significant changes across all measured parameters in samples originating from mtCB_1_^−/−^ mice, clearly pointing to significant mitochondrial enlargement in the knockout mice. These findings possibly indicate mitochondrial stress or dysfunction, as well as an accumulation or aggregation of these enlarged mitochondria in certain areas.

### 3.5. The Lack of Mitochondrial CB_1_ Leads to Altered Mitochondrial Respiration

Our subsequent experimental objective was to determine if the modified mitochondrial morphology resulting from suppressed mitochondrial CB_1_ expression was also associated with changes in mitochondrial activity. To evaluate this hypothesis, we utilized high-resolution respirometry, applying both short and long protocols, to assess potential alterations in the mitochondrial respiration of *m. gastrocnemius* (GA) fibers isolated from the hind limbs of both genetic backgrounds.

**Figure 5:**
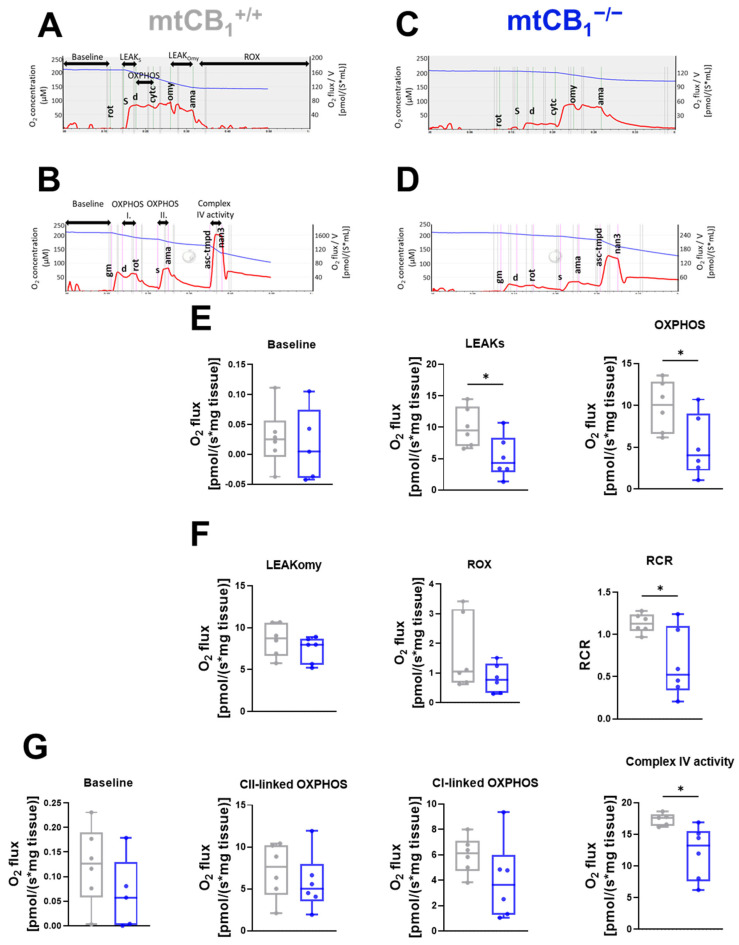
**Mitochondrial function in skeletal muscles is altered following mtCB_1_ deletion.** Representative traces of the short (**A**,**C**) and long (**B**,**D**) treatment protocol for mitochondrial respiration measurement performed on GA muscle fiber bundles. The traces indicate the chamber O_2_ concentrations (blue) and O_2_ consumption (red). (**E**) Baseline, LEAK_S_ state, and oxidative phosphorylation (OXPHOS). (**F**) LEAK_Omy_ states are presented as O_2_ flux (pmol/[s × mg tissue]). Residual oxygen consumption (ROX), and respiratory control ratio (RCR) are plotted as the ratio of OXPHOS and LEAK_Omy_ status. (**G**) Measured baseline, Complex I-linked oxidative phosphorylation (Complex I), Complex II-linked oxidative phosphorylation (Complex II), and Complex IV respiratory activity (Complex IV) are presented as O_2_ flux (pmol/[s × mg tissue]). The box plots demonstrate the median (horizontal line in the box). GA fiber bundles were isolated from both legs of the given animal. *N* = 3 animals/group. * indicates statistical significance at *p* < 0.05.

First, we applied the short respiration protocol to focus on a few key respiratory states and to provide a quick overview of mitochondrial health (Figure 5A,C). This allowed us to determine oxidative phosphorylation (OXPHOS) capacity and leak respiration before and after oligomycin (Omy) application, which inhibits ATP synthesis, resulting in a resting or unphosphorylated state (Figure 5E). Furthermore, routine or baseline respiration, residual oxygen consumption (ROX), and the respiratory control ratio (RCR) were also measured (Figure 5F). Following mtCB_1_ deletion, we observed a significant decline in LEAK_S_ state (10.03 ± 1.28 pmol/s × mg tissue vs. 5.26 ± 1.38* pmol/s × mg tissue, * *p* < 0.05), OXPHOS (9.88 ± 1.25 pmol/s × mg tissue vs. 5.15 ± 1.51* pmol/s × mg tissue, *p* < 0.05), as well as RCR values (0.11 ± 0.004 pmol/s × mg tissue vs. 0.06 ± 0.01* pmol/s × mg tissue, * *p* < 0.05).

This respirometry data suggests reduced OXPHOS and ETC capacity of mtCB_1_^−/−^ mitochondria. The diminished ability of mtCB_1_^−/−^ mitochondria to consume oxygen and thus produce ATP via both Complex I and combined Complex I&II pathways, as well as reduced overall ETC capacity, may directly impact the cell’s energy-generating capabilities.

A more detailed, multi-faceted analysis of mitochondrial respiration can be achieved by systematically introducing a wider range of substrates, un-couplers, and inhibitors, enabling the quantification of numerous respiratory states and control ratios. Hence, baseline and OXPHOS linked to Complex I and II and Complex IV activity were evaluated here using the longer respiration protocol (Figure 5B,D). Among these functional values, only Complex IV showed a change, specifically a significant decrease in the knockout samples (Figure 5G, 17.35± 0.4 pmol/s × mg tissue vs. 12.11 ± 1.71* pmol/s × mg tissue, * *p* < 0.05). A noteworthy observation from the respirometry experiments was the general downward trend across all assessed parameters in the mtCB_1_ knockout.

### 3.6. The Lack of Mitochondrial CB_1_ Leads to Faster Dissipation of Mitochondrial Membrane Potential

**Figure 6:**
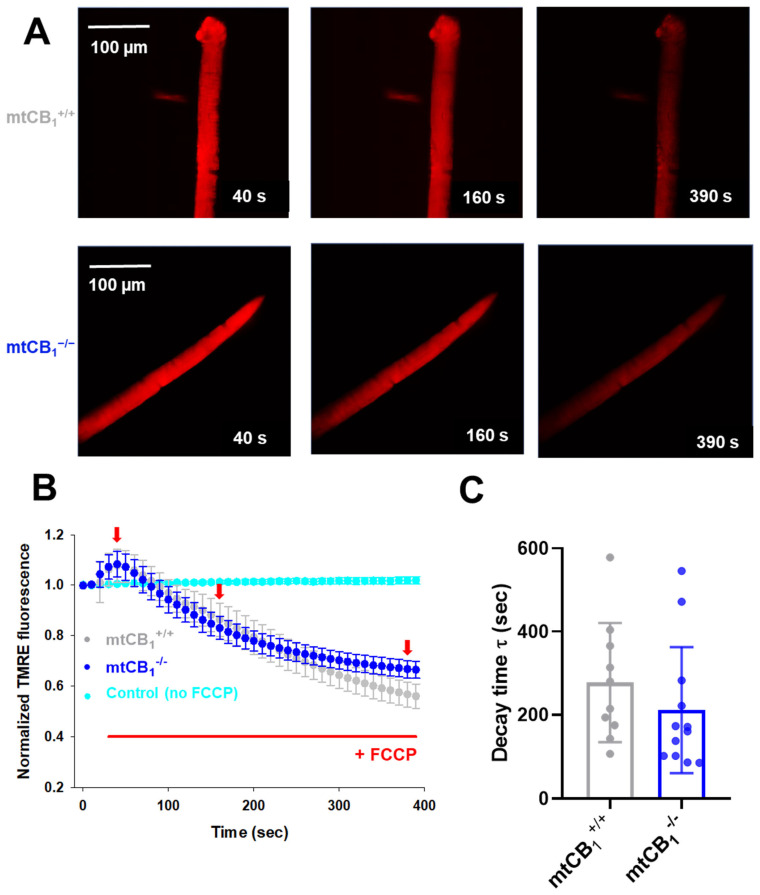
**Faster dissipation of mitochondrial membrane potential** (**Δψ_m_) in mtCB_1_^−/−^ mice.** (**A**) Representative confocal image series of TMRE fluorescence recorded on a single FDB fiber from a mtCB_1_^+/+^ and a mtCB_1_^−/−^ mouse before (40 s) and during the application of 1 µM FCCP (also indicated in panel (**B**) by the red horizontal line). Note the fading over time of the dye fluorescence as the un-coupler poisons the organelle, leading to *Δψ_m_* loss. Scale is 100 µm. (**B**) The averaged normalized TMRE fluorescence decay was faster in mtCB_1_^−/−^ mice. The red arrows indicate the time point when images in panel A were recorded. Data are plotted as means ± SEM. In cyan is the average of 5 control (C57Bl/6) FDB cells, where no FCCP was administered. (**C**) Averaged decay time values were smaller by trend following FCCP administration in the mtCB_1_^−/−^ specimens, indicating faster collapse of mitochondrial membrane potential. *N* = 3/4 mice/group and *n* = 10/12 cells/genotype. Data are plotted as means ± SD.

We delved deeper into the decline in Complex IV activity observed in mtCB_1_^−/−^ mice during respirometry experiments (Figure 5G). In a new set of experiments, we loaded single isolated FDB fibers from both strains with 20 nM TMRE. While continuously scanning the fiber of interest with a confocal microscope, we followed the process of mitochondrial membrane potential (*ΔΨ_m_*) dissipation upon the application of 1 µM FCCP, a well-known un-coupler of the electron transport chain (ETC). Figure 6A presents a few time stamps from a series of representative confocal images recorded on a single cell originating from a mtCB_1_^+/+^ and a mtCB_1_^−/−^ specimen, respectively. Initially (at 40 s) before the un-coupler application, the fiber showed bright, uniform red fluorescence, indicating a high mitochondrial membrane potential (polarized mitochondria). As time passed (at 160 s), the fluorescence intensity appeared to have slightly decreased, suggesting some depolarization. At 390 s into scanning, the fluorescence intensity was noticeably dimmer and more heterogeneous, indicating significant mitochondrial depolarization and loss of membrane potential over time. To quantify the changes, we used exponential fitting for individual cells (like those presented in panel A) to determine the decay time (τ) of *ΔΨ_m_* dissipation. We analyzed τ values from *n* = 10 mtCB_1_^+/+^ and *n* = 12 mtCB_1_^−/−^ cells obtained from 3–4 animals for each genotype. The averaged τ values for both groups are plotted in Figure 6C. Our findings showed that the average decay time for mtCB_1_^+/+^ was τ = 277.65 ± 45.24 s, while that for mtCB_1_^−/−^ was faster by trend at τ = 211.66 ± 43.62 s. This means that the normalized TMRE fluorescence, and thus the mitochondrial membrane potential, dissipated somewhat quicker in the mtCB_1_^−/−^ fibers. To rule out issues like dye bleaching or leakage, we performed control experiments without FCCP application on 5 wild-type C57Bl/6 cells, confirming that there was no significant signal loss (Figure 6B, cyan symbols).

### 3.7. Suppression of Mitochondrial CB_1_ Expression Results in Increased Mitochondrial Calcium Levels

**Figure 7:**
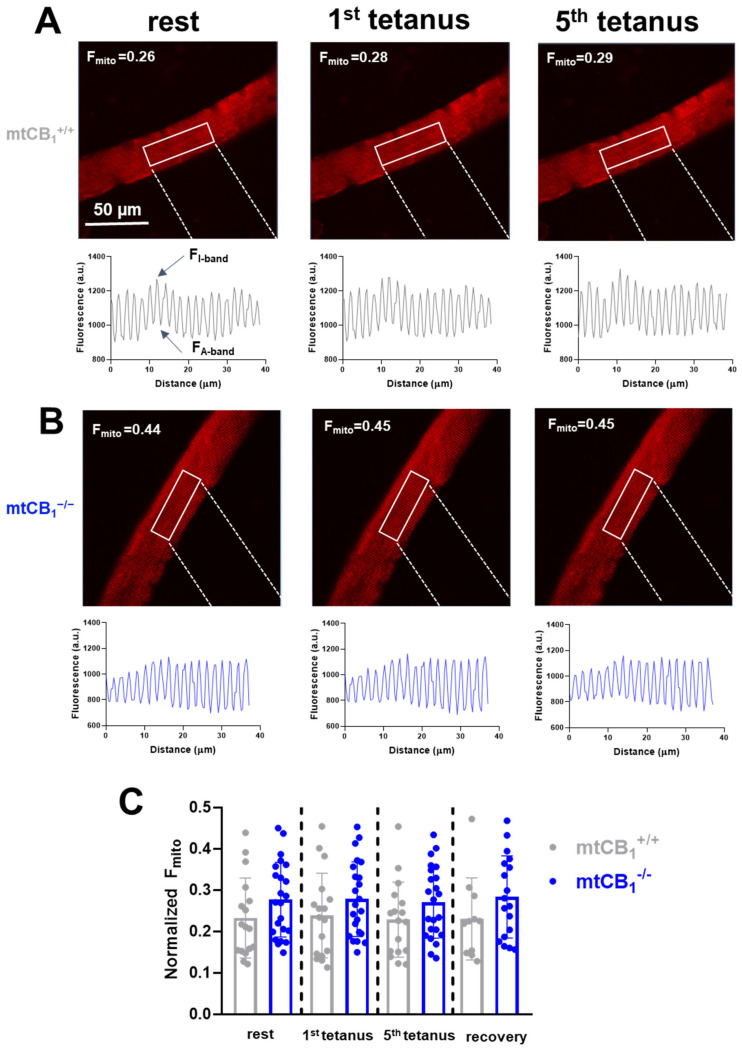
**Mitochondrial fluorescence. Hence, calcium is elevated in mtCB_1_^−/−^ mice in an activity-independent manner.** (**A**,**B**) Representative confocal image series of rhod-2 AM fluorescence recorded on single FDB fibers from a mtCB_1_^+/+^ and a mtCB_1_^−/−^ mouse at rest and following 1 or 5 consecutive tetanic stimulations. The mitochondrial fluorescence intensity profiles were plotted from arbitrarily chosen rectangular areas like the ones highlighted in each image. The normalized mitochondrial fluorescence (F_mito_) was calculated using Equation3 in FDBs from both genotypes. The numbers indicate the respective F_mito_ values. Scale is 50 µm. (**C**) Bar graph representation of the individual mitochondrial fluorescence (F_mito_) data points overlaid at rest, following stimulation, and during recovery, measured 10 min past the last stimulation. Note the differences (albeit statistically non-significant) already at rest of the normalized F_mito_ observed in mtCB_1_^+/+^ and mtCB_1_^−/−^. *N* = 3/4 mice/group and *n* = 17/23 cells/genotype. Data is presented as mean + SD.

It has been proposed that the activation of mtCB_1_Rs affects mitochondrial respiration and ATP production. Given the close link between mitochondrial calcium and these processes, it seemed highly probable that mtCB_1_ receptors might play a role in fine-tuning mitochondrial calcium dynamics [57]. Hence, we assessed the effect of the mtCB_1_R deletion on stimulation-dependent calcium uptake processes by the mitochondria. Normalized mitochondrial fluorescence (F_mito_) was calculated using Equation 3 at rest, following electrical stimulation, and during recovery. A slight, yet progressive increase in F_mito_ values from rest to the 5th tetanus can be observed in the mtCB_1_^+/+^ specimens (Figure 7A). There is very little change in F_mito_ values across the different tetanus conditions in the mtCB_1_^−/−^ mice (Figure 7B). Nevertheless, in the representative examples presented in Figure 7A,B, F_mito_ values in mtCB_1_^−/−^ mice are noticeably higher by trend at rest compared to those in wild-type mice (F_mito_ = 0.26 for mtCB_1_^+/+^ vs. F_mito_ = 0.44 mtCB_1_^−/−^). This may suggest a more “dispersed” or less clustered mitochondrial network in the absence of mtCB_1_ receptors. Further analyzing the data set with individual data points (Figure 7C), one can see that both groups show some variability (scattered dots above the bars) in the fluorescence change to tetanic stimulation, but the overall trend of higher normalized F_mito_ in the mtCB_1_^−/−^ group is clear. As a result, it appears that the suppression of mitochondrial CB_1_ leads to somewhat increased mitochondrial calcium levels as inferred by the rhod-2 fluorescence data.

## 4. Discussion

In this work, employing a systemic mitochondrial CB_1_ deletion murine model, we explored the multifaceted consequences of the suppression of mtCB_1_ expression, primarily impacting muscle force, muscle mitochondrial morphology, respiration, membrane potential, as well as calcium levels.

We replicated the genetic engineering strategy described by Hebert-Chatelain in 2016 [39], which is aimed at interfering with mitochondrial targeting by specifically disrupting only the mitochondrial pool of CB_1_Rs while leaving plasma membrane CB_1_Rs intact (Figure 1A). The strategy involves removing 63 nucleotides (21 amino acids) that have been identified as being responsible for the mitochondrial targeting of the receptor. The exact mutation was published in an elegant study by Soria-Gomez et al. in 2021 [58]. First, to confirm that mtCB_1_Rs were indeed absent in our genetically engineered model, we employed high-resolution STED microscopy, a technique that allowed us to precisely pinpoint the subcellular locations of two separate CB_1_R populations in skeletal muscles (Figure 1B,C). The noticeable rightward shift in the special antibody-labeled CB_1_ receptor fluorescence frequency distribution histogram (Figure 1E) strongly inferred that the mitochondrial CB_1_R pool had been successfully suppressed in the mtCB_1_^−/−^ specimens, while expression of pCB_1_Rs remained chiefly unaltered.

Motor function and coordination were assessed through various tests, and suppression of mtCB_1_ expression did not affect any of the measured parameters, including grip strength, Rota-Rod latency to fall, or wire hang ability (Figure 2). These data suggest that deletion of mtCB_1_ does not modify these capabilities. On the other hand, the global CB_1_ knockout mice were described to show hypoactivity, reduced exploratory behavior, and increased immobility in motor tests, which was correlated with reduced motivation for goal-directed behaviors [59], arguing that motivation is most likely not dependent on mtCB_1_R, but is rather linked to the plasma membrane subpopulation of CB_1_R.

To get a deeper insight into the role of mtCB_1_ on skeletal muscle physiology, we analyzed the in vitro force production in the EDL and SOL muscles of both genotypes. Assessment of tetani and twitches revealed significantly smaller specific normalized force values in the mtCB_1_^−/−^ specimens (Figure 3A–H, Table 1 and Table 2). Further scrutiny of the measured parameters unveiled that the EDL muscles from the mtCB_1_^−/−^ mice exhibited signs of hypertrophy as they were significantly heavier than those from wild-type mice (Table 1), and their cross-sectional area was also significantly greater. This suggests a compensatory mechanism or a dysregulation of muscle growth pathways in the absence of mtCB_1_ (not investigated in this work), even though the specific force is reduced. In the literature, there are clues related to notable enhancements in muscle size and related growth processes following genetic knockout of the CB_1_Rs in skeletal muscles [36]. Furthermore, embryonic and postnatal CB_1_ knockout mice display a greater number of muscle fibers and an increase in muscle fiber diameter compared to wild-type controls [25]. The possible molecular mechanisms behind this process could be (1) enhanced AKT signaling, which could promote anabolic processes; (2) upregulated mTOR activity, which is crucial for muscle protein synthesis; (3) reduced myostatin expression, which is a negative regulator of muscle growth; (4) increased IL-6 secretion, a myokine associated with muscle regeneration and growth. On the other hand, there were no significant differences in contraction kinetics (TTP, HRT, duration). Nevertheless, the force–frequency analysis revealed faster fatigue at higher frequencies in the mtCB_1_^−/−^ specimens after reaching the maximum force output upon stimulation (80 Hz for EDL and 60 Hz for SOL, Figure 3I,J). Noteworthy, in our earlier work, we showed that the muscle-specific inducible CB_1_ knockdown mice (acute and incomplete CB_1_ ablation) displayed altered in vivo and in vitro force generation. The latter displayed similar trends to those described here (see Figure 3C from Singlár et al. 2022 [37]). A change in muscle fiber composition was recently documented by Senese et al. [32]; namely, a decrease in MyoFast (MHCIIb) protein levels and a simultaneous increase in MyoSlow (MHCIb) in the CB_1_ global knock-out model (chronic deletion) was seen. Gonzalez-Mariscal et al. had a similar observation in a conditional skeletal muscle-specific CB_1_^−/−^ mice [36]. The authors drew the conclusion that the observed changes resulted in enhanced physical performance and an overall improvement in whole-body metabolism in mice. Importantly, our data strongly argue that lack of the mitochondrial (and not the plasma membrane) subpopulation of CB_1_R may play a pivotal role in this improvement.

Our in vitro force measurement data suggest that a mitochondria-expressed subset of CB_1_Rs plays a crucial role in regulating muscle strength and possibly muscle size, particularly in fast-twitch fibers. The reduced specific force despite increased size in EDL is a particularly interesting finding, indicating a deficit in the quality or efficiency of force production rather than simply a reduction in muscle mass. Our findings seem to correlate well with earlier data done in cells and mice, showing that the cannabinoid system might orchestrate muscle growth [22]. Furthermore, CB_1_ is known to influence mTOR and AMPK signaling (both known signaling pathways that regulate autophagy) and muscle growth [60]. Whether the increased muscle mass observed in the case of EDL muscles is a direct effect of the suppression of mtCB_1_R or develops secondarily to compensate for the lower normalized force generation of said muscles remains to be elucidated in a follow-up study.

Studies have indicated that the endocannabinoid system can regulate both the integrity and functionality of mitochondria, thereby contributing to the maintenance of cellular energy homeostasis [19,61]. The TEM image analysis we performed here strongly suggests that suppression of mtCB_1_ expression leads to significant morphological abnormalities in muscle mitochondria (Figure 4). Thus, we believe our TEM analysis provides a cellular basis for the observed functional deficits during force measurements. The enlarged mitochondria may contribute to the overall increase in muscle cell volume, or they could be a compensatory hypertrophy where the muscle tries to grow larger to compensate for its functional weakness, even if the individual contractile units are less efficient due to mitochondrial issues. Another aspect worth mentioning is that presumably, the enlarged, swollen, or dysfunctional mitochondria may be less efficient at producing ATP. Moreover, it has been shown that mitochondria become elongated and interconnected in neurons of adult CB_1_R^−/−^ animals, most likely due to an impairment in mitophagy, leading to a significant decline in memory function [62]. Because we observed similar phenomena in the muscles of mtCB_1_^−/−^ animals (i.e., increased mitochondrial area and perimeter; Figure 4), our data favor the idea that the mitochondrial (and not the plasma membrane) subset of CB_1_ may be responsible for maintaining “mitochondrial health”.

The presence of CB_1_ receptors in the mitochondrial outer membrane allows for a direct, fine-tuned regulation of mitochondrial function, including respiration [31,39,43,44]. Since muscle contraction is highly ATP-dependent, the impaired mitochondrial function could directly contribute to reduced force generation. Hence, we set out to assess mitochondrial respiration, which could indirectly provide clues about cellular ATP production. The respirometry data revealed that Complex IV activity is significantly impaired in muscles of adult mtCB_1_^−/−^ animals. Similarly, even with maximal substrate input for both main entry points into the electron transport chain (i.e., Complex I & II), the overall capacity for ATP production is reduced in the mtCB_1_^−/−^ muscles (Figure 5). Moreover, we also observed reduced overall capacity of the electron transport chain itself in mtCB_1_^−/−^ mitochondria, independent of ATP synthesis coupling. This suggests a problem with the respiratory complexes or their efficiency. The reduced respiratory capacity, despite the possibly larger size of mitochondria (seen in TA muscles, Figure 4), suggests that the enlarged mitochondria are dysfunctional, rather than hyper-functional. Their larger size might be a compensatory response to reduced efficiency or a consequence of impaired mitochondrial dynamics (e.g., fusion without proper fission). Earlier studies have found that in global CB_1_ knockout mice, there is an increased oxidative capacity in muscle [32] and liver [63], paired with altered mitochondrial dynamics and mitochondrial quality control. Nevertheless, the impact of a CB_1_ knockout on mitochondrial respiration can vary depending on the tissue and the specific experimental setup.

Generally, acute activation of mtCB_1_ was shown to decrease mitochondrial respiration (e.g., by inhibiting Complex I) [31,39,43,44], implicating that mtCB_1_R-dependent endogenous cannabinoid signaling may protect mitochondria from being “overused” and hence subsequently damaged, e.g., due to increased long-term production of reactive oxygen species [64]. The comprehensive mitochondrial dysfunction identified here may serve as the direct explanation for the previously documented decline in specific force (muscle weakness) across both EDL and SOL muscles in mtCB_1_^−/−^ mice. The substantial ATP demand of muscle contraction means that any impairment in mitochondrial ATP synthesis would directly result in a reduction of contractile force.

To further explore the exact nature of the above mitochondrial dysfunction, we also demonstrated that mitochondrial membrane potential stability is impaired in mtCB_1_^−/−^ muscles (Figure 6). Indeed, mitochondria in mtCB_1_^−/−^ muscle fibers depolarize more rapidly and/or are less able to maintain their membrane potential compared to mtCB_1_^+/+^. A stable and high ***Δψ_m_*** is the driving force for ATP production via ATP synthase. The faster depolarization and reduced ability to maintain ***Δψ_m_*** may directly explain the previously observed reduced oxidative phosphorylation (OXPHOS) capacity and reduced specific force (muscle weakness) in mtCB_1_^−/−^ muscles. If the proton gradient cannot be maintained effectively, ATP synthesis will be compromised, leading to insufficient energy for muscle contraction. The respirometry data with ADP and oligomycin (Figure 5) suggest that the observed depolarization in isolated fibers may be related to processes that consume the proton gradient (like ATP synthesis or even reverse ATPase activity if ATP accumulates or there’s an energy deficit). The faster depolarization in the mtCB_1_^−/−^ suggests a greater susceptibility to these processes, or an intrinsic instability of their inner mitochondrial membrane, or perhaps could be linked to the impaired Complex IV activity.

Mitochondrial calcium is a critical regulator of mitochondrial metabolism (e.g., activating enzymes in the Krebs cycle and oxidative phosphorylation). Moreover, mitochondrial calcium contributes to cellular calcium homeostasis and signaling [3,57]. Altered mitochondrial calcium could impact various calcium-dependent cellular processes, including neurotransmission, muscle contraction, and cell death pathways [65,66,67]. Our present findings suggest that mtCB_1_Rs play a role in regulating or buffering mitochondrial calcium uptake (Figure 7). Specifically, the suppression of mtCB_1_R expression led to an altered basal mitochondrial calcium distribution within muscle fibers, characterized by a higher F_mito_ value, implying a more spread-out or less aggregated mitochondrial network. However, acute muscle activity (tetanic stimulation) did not appear to significantly alter this mitochondrial distribution of the dye fluorescence, at least in our current experimental conditions. Our present data suggest that the suppression of mitochondrial CB_1_R expression may lead to an increase in mitochondrial calcium level by trend under resting conditions. This trend seemed to be maintained throughout the stimulation-induced fluorescence changes. We believe this data could suggest that mtCB_1_Rs possibly act to reduce or at least modulate mitochondrial calcium accumulation in skeletal muscle mitochondria, highlighting their potential role in mitochondrial calcium homeostasis.

## 5. Conclusions

Our findings obtained on the newly generated systemic mtCB_1_-KO mouse model align with the hypothesis that mitochondrial CB_1_ receptors play a critical role in maintaining mitochondrial health and function, which in turn impacts overall muscle performance. Suppression of mtCB_1_ expression leads to mitochondrial dysfunction at a very fundamental level, directly impacting energy production and, consequently, skeletal muscle performance.

## Figures and Tables

**Table 1:** Parameters of twitches and tetani in EDL muscles.*, ** show significant difference from mtCB_1_^+/+^ at *p* < 0.05 and *p* < 0.01, respectively. Data are presented as mean ± SEM.

EDL	TWITCH		TETANUS	
	+/+	−/−	+/+	−/−
**Number of animals**	5	5	5	5
**Number of muscles**	10	9	10	9
**Muscle weight (mg)**	13.67 ± 0.85	16.74 ± 0.45 **		
**Force (mN/mm^2^)**	1.67 ± 0.18	1.12 ± 0.16 *	9.09 ± 1.07	5.33 ± 0.71 *
**TTP (ms)**	31.9 ± 0.9	37.9 ± 3.2	176.8 ± 9.1	177.9 ± 13.8
**HRT (ms)**	27.6 ± 1.4	28.3 ± 1.7	92.2 ± 7.4	93.2 ± 11.1
**Duration (ms)**	262.6 ± 51.7	209.0 ± 31.9	368.7 ± 13.7	362.6 ± 7.3
**CSA (mm^2^)**	0.99 ± 0.08	1.24 ± 0.05 *		

**Table 2:** Parameters of twitches and tetani in SOL muscles. * shows significant difference from mtCB_1_^+/+^ at *p* < 0.05. Data are presented as mean ± SEM.

SOLEUS	TWITCH		TETANUS	
	+/+	−/−	+/+	−/−
**Number of animals**	5	5	5	5
**Number of muscles**	8	9	8	9
**Muscle weight (mg)**	16.14 ± 1.63	18.03 ± 0.65		
**Force (mN/mm^2^)**	1.56 ± 0.30	0.85 ± 0.17 *	9.56 ± 1.48	5.61 ± 0.93 *
**TTP (ms)**	65.3 ± 4.4	69.1 ± 4.7	526.5 ± 3.4	520.0 ± 3.2
**HRT (ms)**	65.5 ± 4.5	78.3 ± 8.9	110.9 ± 4.2	114.8 ± 5.8
**Duration (ms)**	335.3 ± 22.6	364.9 ± 24.2	788.3 ± 16.2	803.1 ± 29.4
**CSA (mm^2^)**	0.95 ± 0.10	1.14 ± 0.07		

## Data Availability

Dataset available on request from the authors.

## Abbreviations

The following abbreviations are used in this manuscript:

AmA	antimycin A
CytC	cytochrome C
Glu	glutamate
Asc-TMPD	ascorbate and N,N,N’,N’-Tetramethyl-p-phenylenediamine dihydrochloride
BSA	bovine serum albumin
cAMP	cyclic AMP
CB_1_R	cannabinoid receptor type 1
CB_2_R	cannabinoid receptor type 2
CSA	cross-sectional area
DMD	Duchenne Muscular Dystrophy
ECC	excitation–contraction coupling
ECS	endocannabinoid system
EDL	extensor digitorum longus
ETC	electron transport chain
FCCP	carbonyl cyanide-p-trifluoromethoxyphenylhydrazone
FDB	flexor digitorum brevis
GA	gastrocnemius
STED	stimulated emission depletion
HRT	half relaxation time
IL-6	interleukin-6
mtCB_1_R	mitochondrially expressed CB_1_ receptor
mTOR	mammalian target of rapamycin
NaN_3_	sodium azide
Omy	oligomycin
OXPHOS	oxidative phosphorylation
OXPHOS I	Complex I-linked oxidative phosphorylation
OXPHOS II	Complex II-linked oxidative phosphorylation
PBS	phosphate-buffered saline
pCB_1_R	plasma membrane CB_1_ receptor
PCR	respiratory control ratio
PKA	protein kinase A
ROI	region of interest
ROS	reactive oxygen species
Rot	rotenone
ROX	residual oxygen consumption
S	succinate
SOL	soleus
TA	tibialis anterior
TMPD	N,N,N’,N’-Tetramethyl-p-phenylenediamine dihydrochloride
TMRE	tetramethylrhodamine methyl ester
TTP	time to peak
