# Correction: Singlár et al. Revealing the Specific Contributions of Mitochondrial CB_1_ Receptors to the Overall Function of Skeletal Muscle in Mice. *Cells* 2025, *14*, 1517

**DOI:** 10.3390/cells15090742

**Published:** 2026-04-22

**Authors:** Zoltán Singlár, Péter Szentesi, Nyamkhuu Ganbat, Barnabás Horváth, László Juhász, Mónika Gönczi, Anikó Keller-Pintér, Attila Oláh, Zoltán Máté, Ferenc Erdélyi, László Csernoch, Mónika Sztretye

**Affiliations:** 1 Department of Physiology, Faculty of Medicine, University of Debrecen, 4032 Debrecen, Hungary; singlar.zoltan@med.unideb.hu (Z.S.); szentesi.peter@med.unideb.hu (P.S.); nyamkhuu.ganbat@med.unideb.hu (N.G.); gonczi.monika@med.unideb.hu (M.G.); olah.attila@med.unideb.hu (A.O.); csl@edu.unideb.hu (L.C.); 2 HUN-REN Cell Physiology Research Group, University of Debrecen, 4032 Debrecen, Hungary; 3 Doctoral School of Molecular Medicine, University of Debrecen, 4032 Debrecen, Hungary; 4 Department of Biochemistry, Albert Szent-Györgyi Medical School, University of Szeged, 6720 Szeged, Hungary; horvath.barnabas@med.u-szeged.hu (B.H.); keller.aniko@med.u-szeged.hu (A.K.-P.); 5 Centre of Excellence for Interdisciplinary Research, Development and Innovation, University of Szeged, 6720 Szeged, Hungary; 6 Institute of Surgical Research, Albert Szent-Györgyi Medical School, University of Szeged, 6720 Szeged, Hungary; juhasz.laszlo.1@med.u-szeged.hu; 7 Medical GeneTechnology Unit, HUN-REN Institute of Experimental Medicine, 1083 Budapest, Hungary; matez@koki.hu (Z.M.); erdelyi@koki.hu (F.E.)

**Missing Citation**

In the original publication [1], Ref. [2] was not cited. The citation has now been inserted in the Discussion, 2nd paragraph, and should read:

We replicated the genetic engineering strategy described by Hebert-Chatelain in 2016 [39], which is aimed at interfering with mitochondrial targeting by specifically disrupting only the mitochondrial pool of CB_1_Rs while leaving plasma membrane CB_1_Rs intact (Figure 1A). The strategy involves removing 63 nucleotides (21 amino acids) that have been identified as being responsible for the mitochondrial targeting of the receptor. The exact mutation was published in an elegant study by Soria-Gomez et al. in 2021 [58].

**Text Correction**

There was an error in the original publication. We incorrectly claimed that we investigated the first-ever global mitochondrial CB1 knockout mouse model. 

A correction has been made to the last paragraph of the Introduction:

In the current study we created a global mitochondrial CB_1_ receptor mouse model. By using a combination of genetic and pharmacological approaches, we report a key role of mtCB_1_Rs in skeletal muscle physiology. Our findings offer novel insights into the complex involvement of mtCB_1_Rs in ECS-mediated regulation of skeletal muscle contraction, force generation, mitochondrial morphology, and energetics.

A correction has been made to the first paragraph of the Discussion:

In this work, employing a systemic mitochondrial CB_1_ deletion murine model, we explored the multifaceted consequences of the suppression of mtCB_1_ expression, primarily impacting muscle force, muscle mitochondrial morphology, respiration, membrane potential, as well as calcium levels.

A correction has been made to the first paragraph of the Conclusion:

Our findings obtained on the newly generated systemic mtCB_1_-KO mouse model align with the hypothesis that mitochondrial CB_1_ receptors play a critical role in maintaining mitochondrial health and function, which in turn impacts overall muscle performance. Suppression of mtCB_1_ expression leads to mitochondrial dysfunction at a very fundamental level, directly impacting energy production and, consequently, skeletal muscle performance.

**References**

Added Reference 58: Soria-Gomez, E.; Pagano Zottola, A.C.; Mariani, Y.; Desprez, T.; Barresi, M.; Río, I.B.-D.; Muguruza, C.; Bon-Jego, M.L.; Julio-Kalajzić, F.; Flynn, R. Subcellular specificity of cannabinoid effects in striatonigral circuits. *Neuron* **2021**, *9*, 1513–1526.e11. https://doi.org/10.1016/j.neuron.2021.03.007. With this correction, the order of some references has been adjusted accordingly.

The authors state that the scientific conclusions are unaffected. This correction was approved by the Academic Editor. The original publication has also been updated.
